# Genotype-Encoded UV Sensitivity in iPSC-Derived Human Melanocytes Reveals MX2 as a Physiological Amplifier of p53/p38-Mediated DNA Damage Signaling

**DOI:** 10.3390/ijms27062617

**Published:** 2026-03-12

**Authors:** Eric Ramirez-Salazar, Ana Slipicevic, Marina Juraleviciute, Ling Li, Mark Harland, Sally O’Shea, Sinead Field, Julia Newton-Bishop, Meenhard Herlyn

**Affiliations:** 1 The Wistar Institute, Philadelphia, PA 19104, USA; slipicevic@yahoo.no (A.S.); lingli@wistar.org (L.L.); 2 Department of Pathology, Oslo University Hospital, Norwegian Radium Hospital, 0379 Oslo, Norway; marinaj_88@yahoo.com; 3 Laser Research Center, Faculty of Physics, Vilnius University, LT-01513 Vilnius, Lithuania; 4 Leeds Institute of Rheumatic and Musculoskeletal Medicine (LIRMM), University of Leeds, Leeds LS2 9JT, UK; m.harland@leeds.ac.uk (M.H.); sally_o_shea@outlook.com (S.O.); sinead.field@hse.ie (S.F.); j.a.newton-bishop@leeds.ac.uk (J.N.-B.)

**Keywords:** induced pluripotent stem cells, iPSC-derived melanocytes, ultraviolet radiation, MC1R variants, DNA damage response, MX2, p53 signaling, new approach methodologies

## Abstract

Ultraviolet (UV) radiation induces DNA damage and oxidative stress in melanocytes, shaping pigmentation phenotypes and elevating photocarcinogenesis risk. Human models that capture donor-linked genetic determinants of UV sensitivity remain limited. Here, we establish a genotype-informed UV response model using induced pluripotent stem cell (iPSC)-derived melanocytes from donors carrying defined *MC1R* variants. Differentiated cells recapitulated melanocytic morphology, marker expression, and pigmentation consistent with donor sun-sensitivity traits. Following narrowband UVB exposure, melanocyte lines with higher UV sensitivity showed reduced survival, prolonged checkpoint activation, and CPD-associated DNA damage signaling dynamics. Mechanistic analysis suggests that the interferon-regulated GTPase MX2 is associated with amplification of UV-induced p53 and p38 activation while promoting apoptosis independently of AKT. These findings support MX2 as a physiological enhancer of DNA damage signaling in normal melanocytes, distinct from its interferon-mediated role in melanoma. Our study provides a human-relevant platform linking pigmentation genotype to UV resilience and supports iPSC-derived systems as new approach methodologies (NAMs) for mechanistic and translational phototoxicology.

## 1. Introduction

Ultraviolet (UV) radiation is a pervasive environmental stressor that inflicts direct and indirect damage on skin cells through cyclobutane pyrimidine dimers (CPDs), 6-4 photoproducts, and reactive oxygen species (ROS) [1,2]. ROS exacerbate cellular injury by oxidizing lipids and nucleic acids, activating the Nrf2-Keap1 antioxidant pathway, and modulating apoptosis via MAPK cascades [3,4,5]. The efficiency of these repair and detoxification mechanisms varies among individuals and is strongly influenced by pigmentation genotype.

The melanocortin-1 receptor (MC1R) governs melanogenesis, the eumelanin/pheomelanin ratio, and cAMP/PKA-CREB signaling after UV exposure [6,7]. Functional *MC1R* variants favor eumelanin synthesis and activate p53-mediated DNA repair, whereas loss-of-function alleles, common in red-hair and fair-skin phenotypes, impair nucleotide excision repair (NER) and increase melanoma susceptibility [8,9]. Beyond melanin photoprotection, MC1R signaling intersects with ATR/CHK1 checkpoint control and antioxidant responses, shaping cellular resilience to UV-induced DNA lesions [10,11].

Beyond its direct mutagenic effects, ultraviolet (UV) radiation also elicits a robust inflammatory and innate immune response in the skin, characterized by activation of interferon signaling, stress-activated kinases, and pro-apoptotic pathways. These UV-induced responses play a critical role in shaping melanocyte fate after photodamage and have been increasingly implicated in melanoma initiation and progression. Recent studies highlight that inflammatory signaling triggered by UV exposure can modulate DNA damage resolution, immune surveillance, and tumor-promoting microenvironments in the skin [12]. However, how these stress and innate immune pathways intersect with pigmentation genotype to define melanocyte UV sensitivity remains incompletely understood.

Traditional studies rely on primary or transformed melanocytes, which are limited by availability and donor variability. Induced pluripotent stem cell (iPSC) technology enables reprogramming of fibroblasts from genetically defined donors into melanocytes, preserving pigmentation and repair traits [13,14]. Single-cell and transcriptomic analyses validate iPSC-derived melanocytes as faithful models of epidermal pigmentation and photobiology [15,16]. These systems also permit exploration of paracrine interactions with keratinocytes via α-melanocyte-stimulating hormone (α-MSH), endothelins, and stem cell factor (SCF), which modulate melanocyte UV responses [17,18,19].

At the molecular level, the interferon-inducible GTPase MX2 regulates antiviral defense and cell-cycle control [20,21,22,23]. In melanoma, MX2 influences therapeutic response and immune signaling through STAT1, XAF1, and p53 networks [22,23,24,25]. However, its physiological role in normal melanocytes and in the DNA damage response (DDR) to UV stress remains unclear. Preliminary evidence suggests MX2 integrates interferon, DDR, and apoptotic pathways via p38 and p53 crosstalk [25]. Elucidating this axis is critical for linking pigmentation genotype to stress signaling and disease risk.

Here, we establish an iPSC-based human melanocyte model to investigate how pigmentation genotype shapes UV-induced DNA damage responses. Using donor-associated lines carrying distinct MC1R variants, this study examines the relationship between genetic background, checkpoint activation, and repair fidelity. We also explore the role of the interferon-inducible factor MX2 in modulating UV stress responses. Together, these analyses provide a mechanistic framework for genotype-encoded UV sensitivity and support the relevance of iPSC-based NAMs for photobiology research [26,27].

## 2. Results

### 2.1. Generation, Validation, and Selection of Patient-Derived iPSC Lines 

To establish a genetically diverse iPSC resource for modeling pigmentation biology and UV responses, dermal fibroblasts from melanoma patients and healthy donors were reprogrammed using Sendai virus vectors expressing OCT4, SOX2, KLF4, and c-MYC as originally described by Yamanaka and colleagues [27] and later adapted into non-integrating Sendai virus systems for footprint-free reprogramming [28]. Across all donors, we generated a panel of 28 human iPSC lines, each expanded as 4-5 clonal colonies and subjected to standardized pluripotency quality-control (QC) assessments (Supplementary Table S1). Lines passing RT-qPCR, TRA-1-60/SSEA-4 flow cytometry, and morphological criteria (Supplementary Figure S1) were further evaluated using the TaqMan hPSC Scorecard (see QC summary in Supplementary Table S1). Lines exhibiting unstable morphology or suboptimal pluripotency signatures were excluded from downstream work.

From this larger cohort, we prioritized a subset of iPSC lines with fully documented MC1R genotype, normal karyotype, and high-quality pluripotency profiles for melanocyte differentiation. These included donors Leeds003, Leeds004, Leeds006, Leeds007, Leeds008, and Leeds009, which consistently performed robustly across QC assays. Additional successfully reprogrammed lines (e.g., GM09943, GM00671-6, Wi38, FF160, FH202, FH217, FH310) were not advanced due to incomplete metadata, limited fibroblast availability, or inconsistent melanocyte yield.

Together, these data define the full reprogramming pipeline supporting this project and the criteria used to select the subset of iPSC lines for melanocyte differentiation (Supplementary Table S1).

### 2.2. Generation and Characterization of iPSC-Derived Melanocytes with Patient-Specific Pigmentation Genotypes

**Figure 1:**
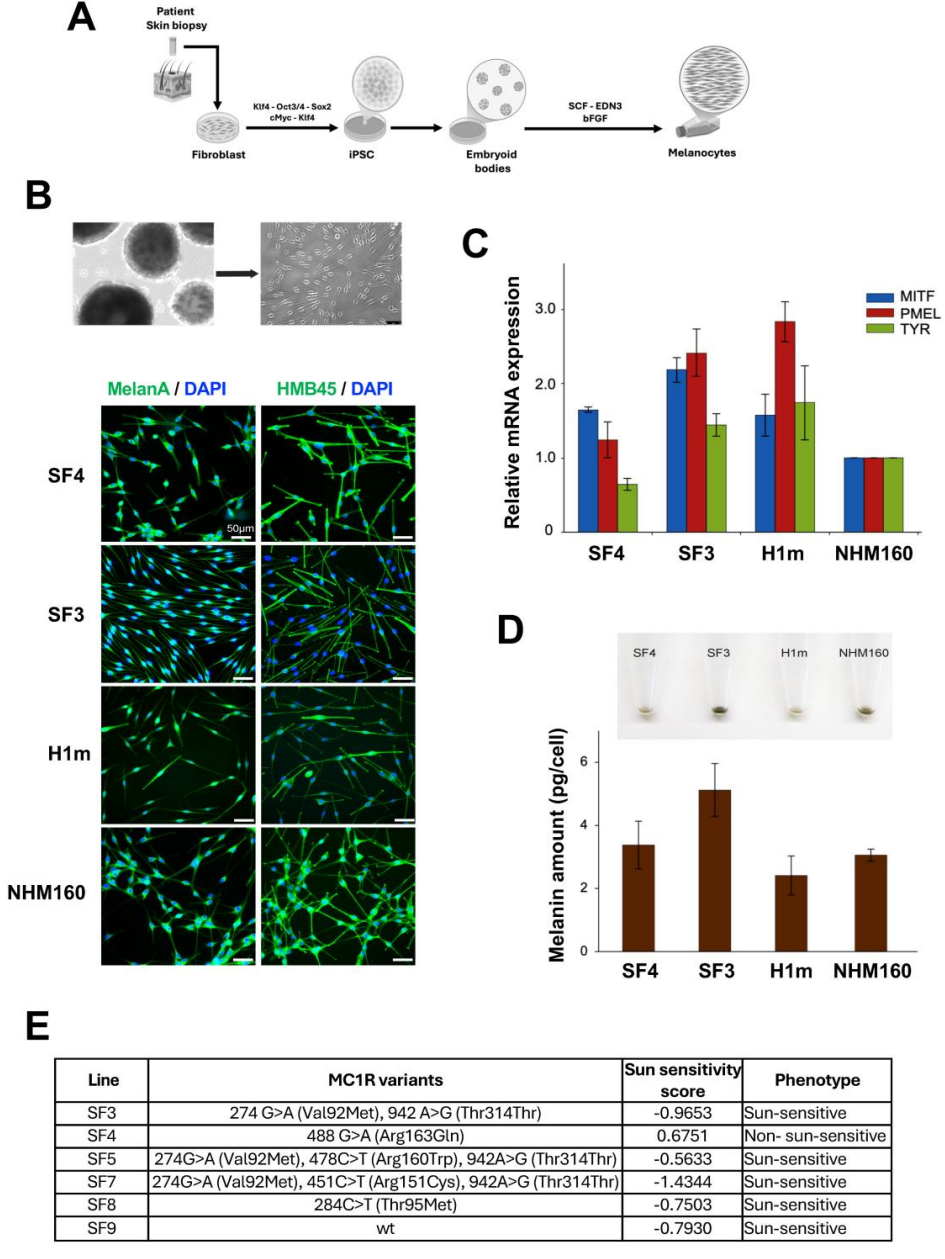
Molecular, morphological, and phenotypic characterization of patient-derived melanocyte lines with distinct sun-sensitivity profiles. (**A**) Schematic representation of the experimental workflow. Skin biopsies from patients were reprogrammed into induced pluripotent stem cells (iPSCs) using OCT3/4, SOX2, KLF4, and c-MYC. iPSCs were subsequently differentiated into melanocytes through exposure to SCF, EDN3, and bFGF. (**B**) Representative phase-contrast micrographs showing the morphology of embryoid bodies (EBs) and differentiated melanocytes derived from iPSCs. Differentiated melanocytes exhibit characteristic dendritic morphology. Immunofluorescence staining of melanocyte-specific markers MelanA and HMB45 in Leeds3, Leeds4, H1m, and NHM160 cell lines. Nuclei were counterstained with DAPI. Scale bar: 50 μm. (**C**) Relative mRNA expression levels of melanocyte lineage genes MITF, PMEL, and TYR in Leeds3, Leeds4, H1m, and NHM160, as determined by quantitative RT-PCR. Expression values are normalized to housekeeping genes. (**D**) Quantification of intracellular melanin content (pg/cell) across the same cell lines, revealing differences in pigment production capacity. (**E**) Summary of MC1R gene variants identified in each cell line, along with corresponding sun-sensitivity scores and phenotypic classification. Lines Leeds3, Leeds4, Leeds5, Leeds7, Leeds8, and Leeds9 exhibit distinct MC1R polymorphisms and sun-sensitivity profiles, which correlate with pigmentation phenotypes. Notably, total melanin content does not correlate strictly with UV resistance, consistent with the known effects of MC1R variants on melanocyte signaling and redox balance rather than pigment quantity alone. H1m and NHM160 cells were included as standardized stem cell-derived and primary melanocyte reference controls, respectively.

Figure 1 provides an overview of the generation and baseline characterization of iPSC-derived melanocytes from donors with distinct MC1R genotypes and pigmentation phenotypes. This figure is intended to document melanocyte identity, pigmentation capacity, and genetic background rather than to serve as a direct functional comparison of UV sensitivity across all lines. The selection of cell lines shown in panels B–D reflects representative iPSC-derived melanocyte lines used for morphological and pigmentation characterization. Leeds9, which carries a wild-type MC1R allele, was included as a genetic reference line but was not displayed in all panels due to space constraints. Notably, sun-sensitivity classifications are based on donor-reported pigmentation traits and may not strictly align with the MC1R genotype alone. Fibroblasts from donors carrying distinct MC1R pigmentation genotypes were reprogrammed into iPSCs and differentiated into melanocytes following a standardized protocol (Figure 1A). The resulting cells exhibited characteristic dendritic morphology and expressed melanocytic markers MelanA and HMB45, confirmed by immunofluorescence (Figure 1B). Additional melanocytic markers, including S100, Mel-5 (TYRP1), and dual-marker combinations, were validated in representative lines (Supplementary Figure S2). Quantitative RT-PCR demonstrated robust expression of the lineage-specific genes MITF, PMEL, and TYR, comparable to that in normal human melanocytes (NHM160) (Figure 1C), consistent with previous reports [12,13,14]. Measurement of melanin content revealed variable pigmentation levels across donor lines, correlating with their MC1R genotypes and reported sun-sensitivity scores (Figure 1D,E; Supplementary Table S2). This genotype–phenotype relationship is consistent with previous work showing that pigmentation pathway activity, including MITF-dependent melanogenic programs, modulates UV resilience in human melanocytes [29].

The iPSC-derived melanocyte lines analyzed in this study were selected to capture a range of pigmentation genotypes and donor-reported sun-sensitivity traits; however, the distribution of sun-sensitive versus sun-insensitive lines was not intended to be balanced for population-level comparisons.

A detailed summary of melanocyte differentiation outcomes for each selected Leeds line, including morphology, pigmentation level, marker expression, and culture timeline, is provided in Supplementary Table S2. These data document line-to-line variability among the Leeds iPSCs and justify their selection for subsequent mechanistic analyses.

Prior to differentiation, pluripotency was verified by flow cytometry and immunostaining for TRA-1-60 and SSEA-4, alongside high mRNA levels of Nanog, Oct4, and Sox2 (Supplementary Figure S1), confirming successful reprogramming. Collectively, these findings establish a reproducible platform for generating melanocytes that preserve donor-linked pigmentation traits, enabling mechanistic studies of UV response in a genetically defined context. For experimental benchmarking, two reference melanocyte populations were included throughout the study. H1m cells represent melanocytes derived from a well-characterized human embryonic stem cell line and serve as a standardized pluripotent stem cell-derived melanocyte reference. NHM160 cells correspond to primary normal human melanocytes and were used as a physiological benchmark for baseline pigmentation, DNA damage response, and UV sensitivity. These control lines were not intended for genotype-based comparisons but rather to provide technical and biological context for interpreting UV response phenotypes observed in donor-derived iPSC melanocytes.

### 2.3. Donor-Associated UV Sensitivity and Cell-Cycle Checkpoint Activation 

**Figure 2:**
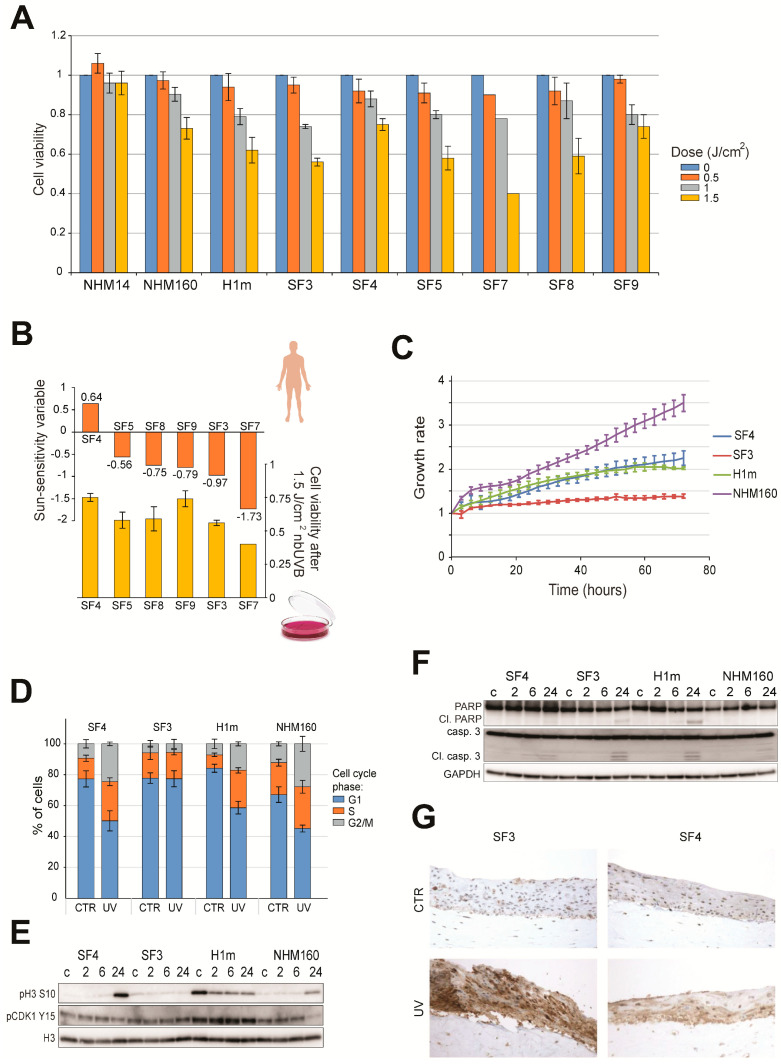
Donor-linked UVB sensitivity is associated with checkpoint activation and apoptosis in iPSC-derived melanocytes. (**A**) Viability of iPSC-derived and normal human melanocytes 72 h after exposure to increasing doses of narrowband UVB (nbUVB; 0–1.5 J/cm^2^). Data represent mean ± SE of three independent experiments. (**B**) Comparison between in vitro viability of melanocytes 72 h after 1.5 J/cm^2^ nbUVB exposure and the corresponding patient’s sun-sensitivity scores derived from self-reported pigmentation traits. A trend between donor-reported sun sensitivity and UV-induced reduction in viability is observed. (**C**) Growth rate assessment of melanocyte lines using a confluence-based assay with the IncuCyte™ FLR live-cell imaging system. Cell confluence was normalized to the initial time point. Data represent mean ± SE of *n* = 3. Ld: Leeds. (**D**) Cell-cycle distribution of melanocytes 24 h post-UVB exposure (1.5 J/cm^2^), evaluated by flow cytometry using propidium iodide staining. Bar graphs show the percentage of cells in G1, S, and G2/M phases (mean ± SE, *n* = 3). (**E**) Immunoblot analysis of G2/M phase-specific markers phospho-Histone H3 (Ser10) and phospho-CDK1 (Tyr15) at 2, 6, and 24 h post-UVB exposure. Checkpoint marker phosphorylation is shown for representative lines and is most pronounced in the highly proliferative H1m cells. Total Histone H3 was used as a loading control. (**F**) Western blot analysis of apoptosis markers, cleaved caspase-3, and cleaved PARP at 2, 6, and 24 h post-UVB exposure. GAPDH was used as a loading control. Representative immunoblots are shown for the indicated time points post-UV irradiation, with apoptotic marker cleavage most clearly detectable at 24 h. (**G**) Leeds3 and Leeds4 melanocytes were incorporated into 3D skin reconstructs, irradiated with 300 mJ/cm^2^ UV, and analyzed 48 h later by immunohistochemistry for cleaved caspase-3 to assess apoptosis in a tissue context. Staining reflects an integrated epidermal response within the reconstructed tissue and is not intended to distinguish melanocyte- versus keratinocyte-specific apoptotic signaling. H1m and NHM160 cells were included as standardized stem cell-derived and primary melanocyte reference controls, respectively.

To determine whether donor genotype influences UV stress resilience, iPSC-derived melanocytes were exposed to increasing doses of narrowband UVB (0–1.5 J/cm^2^). All lines exhibited a dose-dependent decrease in viability; however, survival differed markedly by MC1R genotype (Figure 2A,B). Importantly, the extent of viability loss observed across melanocyte lines appeared broadly consistent with donor-reported sun sensitivity, with sun-sensitive lines showing a steeper apparent decline in survival compared to sun-resistant lines across increasing UV doses. Because the present study includes a limited number of donor-derived melanocyte lines, this observation is presented as a qualitative trend rather than a formal statistical correlation. UV-sensitive lines (Leeds3, H1m) exhibited detectable activation of apoptotic markers, as evidenced by cleaved caspase-3 and PARP detected at 24 h post-irradiation (Figure 2F), whereas resistant lines (Leeds4, NHM160) showed weaker apoptotic signaling at this time point. Notably, NHM14 primary melanocytes did not exhibit a pronounced dose-dependent decrease in viability under the UV conditions tested, consistent with a higher intrinsic UV tolerance and efficient stress-adaptive responses previously reported in certain primary melanocyte populations. Cell-cycle analysis revealed phosphorylation of histone H3 (Ser10) and CDK1 (Tyr15), indicating G2/M checkpoint engagement following UV exposure (Figure 2D,E). Notably, phosphorylation of histone H3 (Ser10) and CDK1 (Tyr15) was clearly detectable in H1m cells following UV exposure, whereas no comparable phosphorylation signal was observed in Leeds3 under the conditions tested (Figure 2E). Flow cytometry analysis revealed that three melanocyte lines displayed a comparable redistribution of cell-cycle phases following UV exposure, whereas Leeds3 remained largely unchanged under the same experimental conditions (Figure 2D).

Additionally, 3D skin reconstructs containing donor-derived melanocytes exhibited differential patterns of UV-associated epidermal stress staining following irradiation (Figure 2G). Because constructs lacking melanocytes were not included, and staining does not distinguish individual cell types, these observations are interpreted as tissue-level epidermal responses rather than melanocyte-specific apoptosis. This experiment was included to assess whether donor-dependent response patterns observed in monolayer cultures remain detectable within a stratified epidermal environment. Collectively, these findings indicate that genotype-associated differences in UV response can be explored under controlled in vitro conditions using iPSC-derived melanocytes within the scope of this mechanistic proof-of-concept study.

Importantly, this comparison was designed as a genotype-informed mechanistic analysis rather than a population-level assessment, and the limited number of melanocyte lines reflects the availability of well-characterized donor material within the experimental framework of this study.

### 2.4. UV-Induced DNA Damage Signaling and Repair Efficiency 

**Figure 3:**
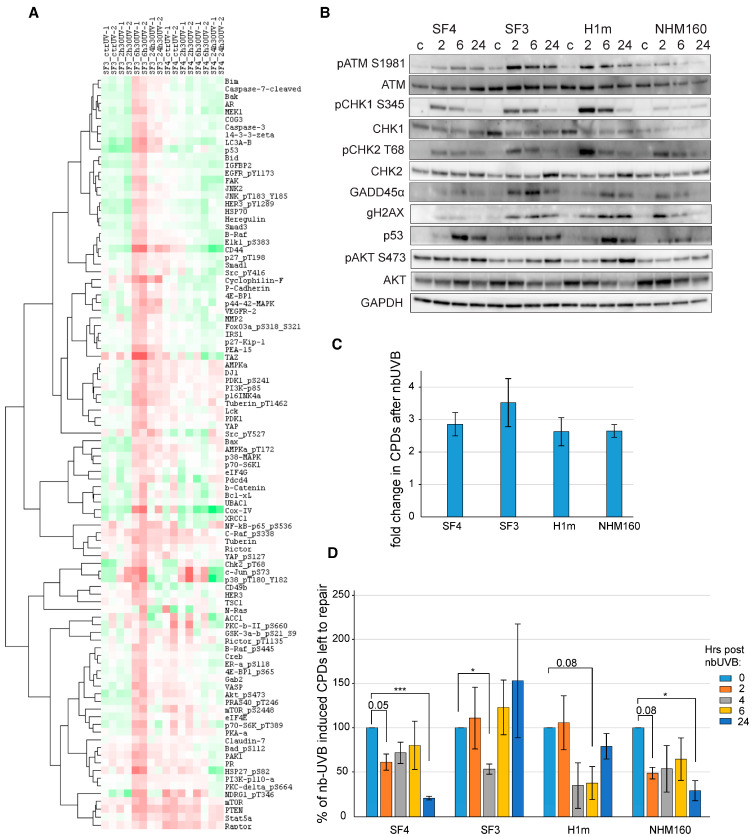
Genotype-associated DNA damage signaling dynamics and CPD processing in iPSC-derived melanocytes. (**A**) Heatmap illustrating differentially activated signaling pathways in Leeds3 and Leeds4 melanocytes at 2, 6, and 24 h after 1.5 J/cm^2^ narrowband UVB (nbUVB) exposure, assessed by reverse phase protein array (RPPA), highlighting pathways involved in DNA damage response, apoptosis, and cell-cycle regulation. Red indicates increased protein/phosphoprotein levels relative to control, whereas green indicates decreased levels. Ld: Leeds. (**B**) Validation of RPPA results by immunoblotting for key DNA damage response proteins, including phosphorylated ATM (Ser1981), CHK1 (Ser345), CHK2 (Thr68), γH2AX, GADD45α, and p53, with GAPDH as loading control. (**C**) Quantification of cyclobutane pyrimidine dimers (CPDs) immediately after nbUVB exposure in Leeds3, Leeds4, H1m, and NHM160 melanocytes using ELISA (mean ± SE, *n* = 3). (**D**) Time-course analysis of CPD repair at 2, 4, 6, and 24 h post-irradiation, expressed as percentage of unrepaired lesions relative to initial damage. Statistical significance was determined by a one-sample *t*-test (* *p* < 0.05, *** *p* < 0.001). H1m and NHM160 cells were included as standardized stem cell-derived and primary melanocyte reference controls, respectively.

To investigate genotype-dependent DNA damage response (DDR), melanocytes were irradiated with 1.5 J/cm^2^ nbUVB and collected at defined intervals (2–24 h) (Figure 3A–D). Figure 3A summarizes reverse phase protein array (RPPA) profiling performed on whole-cell lysates obtained from UV-irradiated melanocytes at the indicated time points. All RPPA measurements were performed on equalized protein inputs derived from whole-cell lysates, ensuring comparability across UV conditions and collection time points.

Protein abundance values were normalized and median-centered relative to untreated controls, allowing comparison of pathway activation independently of variations in cell number or protein loading. Immunoblotting revealed rapid phosphorylation of ATM, CHK1, and CHK2 within 2 h (Figure 3B), confirming activation of canonical DDR pathways [4,16]. Notably, phosphorylation of AKT (S473) was detectable at baseline in Leeds4 melanocytes and persisted after UV exposure, suggesting a higher basal pro-survival signaling tone in this UV-resistant line rather than a UV-induced activation event. However, UV-sensitive lines exhibited sustained γH2AX and GADD45α accumulation up to 24 h, indicating incomplete lesion repair. This pattern parallels recent UVB studies in human skin-derived cell models showing that persistent γH2AX retention and delayed DDR resolution strongly predict apoptotic commitment [30,31].

Phosphorylation of AKT (S473) displayed distinct temporal dynamics across melanocyte lines (Figure 3B), consistent with checkpoint adaptation and survival signaling described in UV-exposed skin cells. CPD levels were quantified by ELISA immediately after nbUVB exposure (Figure 3C) and followed over time to assess CPD processing dynamics within each melanocyte line (Figure 3D). Intermediate time-point variations were not interpreted mechanistically and do not affect the within-line assessment of CPD-associated dynamics. These observations are consistent with previously described associations between DNA damage signaling dynamics and UV response phenotypes in melanocytes [9,16].

Analysis of the RPPA dataset highlighted differential regulation of interferon- and stress-associated signaling pathways across melanocyte lines (Figure 3A). Based on prior biological evidence linking MX2 to interferon signaling and melanoma biology, we prioritized MX2 as a hypothesis-driven candidate for further mechanistic investigation in the context of UV-induced DDR (Figure 3A). Although MX2 was not uniquely highlighted by RPPA profiling, it was prioritized for further investigation based on its established links to stress signaling, interferon responses, and melanoma biology.

### 2.5. MX2 Expression Is Associated with UV-Induced Stress Signaling in Melanocytes 

**Figure 4:**
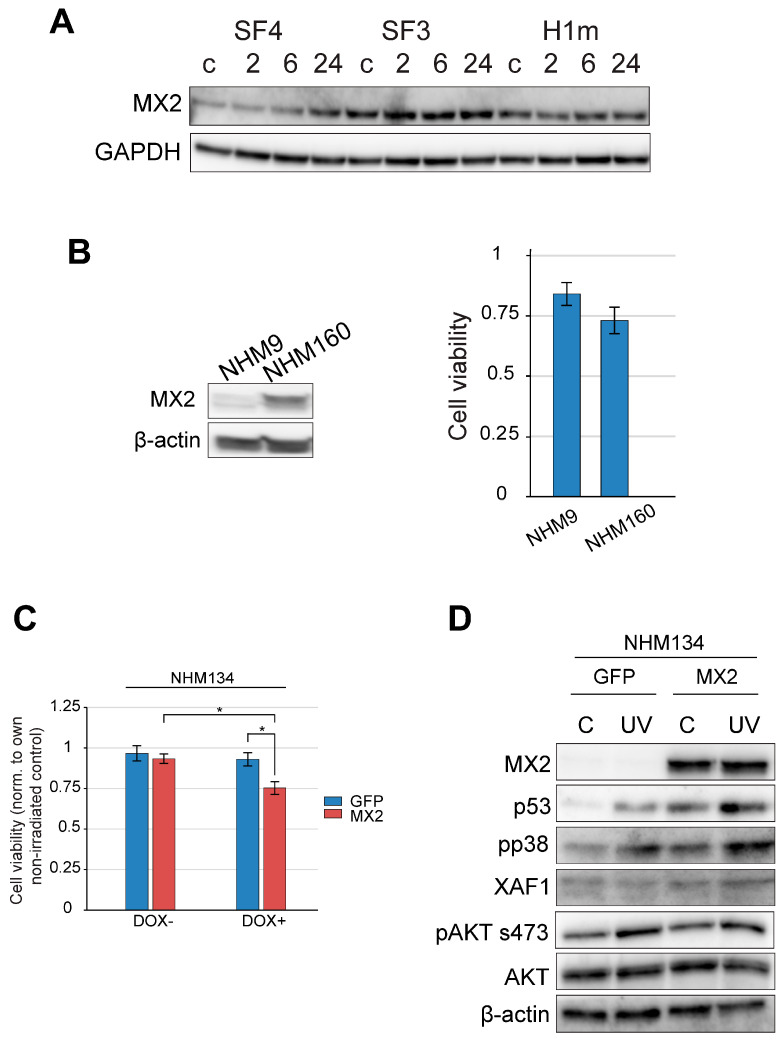
MX2 amplifies UV-induced stress signaling and reduces melanocyte survival via an AKT-independent mechanism. (**A**) Immunoblot analysis of MX2 expression in iPSC-derived melanocytes (Leeds3, Leeds4, H1m) at 2, 6, and 24 h after exposure to 1.5 J/cm^2^ narrowband UVB (nbUVB), with GAPDH as loading control. (**B**) MX2 protein levels in normal human melanocytes NHM9 and NHM160 (left panel) and corresponding cell viability 72 h after 1.5 J/cm^2^ nbUVB irradiation (right panel). Data represent mean ± SE of *n* = 3. (**C**) NHM134 melanocytes were treated with 500 ng/mL doxycycline to induce GFP or MX2 expression 24 h prior to nbUVB exposure (1.5 J/cm^2^), and cell viability was assessed 72 h post-irradiation. Data represent mean ± SE of *n* = 3. Statistical significance was determined by one-way ANOVA followed by Tukey’s multiple comparison test (* *p* < 0.05). (**D**) Immunoblot analysis of NHM134 melanocytes treated as in (**C**), harvested 24 h after nbUVB exposure, and evaluated for MX2, p53, phospho-p38, XAF1, and phospho-AKT (Ser473) expression, with β-actin as loading control.

Given previous reports implicating MX2 in interferon responses and melanoma progression [22,23,24], we examined its role in UV-induced stress signaling in non-transformed melanocytes (Figure 4A–D). For these experiments, additional primary melanocyte lines (NHM9 and NHM160) were included as independent validation models, while NHM134 cells were engineered with a doxycycline-inducible MX2 expression system with GFP as a control to enable controlled assessment of MX2-dependent signaling responses. These melanocyte lines represent defined pigmentation and MC1R backgrounds consistent with those analyzed in earlier sections, ensuring that MX2-associated signaling responses were evaluated within the same genotype-informed framework of UV sensitivity.

Basal MX2 expression varied across donor lines and positively correlated with UV sensitivity and p53 activation (Figure 4A,B). UV exposure induced further MX2 upregulation in sensitive lines, coinciding with strong phosphorylation of p38 and p53 (Ser15) (Figure 4B,D).

Functional perturbation studies in melanoma cells revealed that ectopic MX2 overexpression augmented UV-induced apoptosis and reduced cell viability (Supplementary Figure S3), whereas GFP controls exhibited higher survival. Importantly, MX2 modulation did not significantly alter AKT phosphorylation, suggesting a p53/p38-dependent but AKT-independent mechanism of stress amplification. These results support MX2 as a potential physiological enhancer of UV-induced apoptosis and checkpoint signaling in human melanocytes, distinct from its interferon-mediated role in melanoma [22,23,32], highlighting its potential as a modulator of phototoxic stress and pigmentation-related DNA repair capacity.

## 3. Discussion

This study presents a genotype-driven model of human UV sensitivity using iPSC-derived melanocytes from donors with defined MC1R pigmentation variants. The results demonstrate that UV responses are influenced by donor genetic background and are recapitulated in patient-derived melanocytes, linking pigmentation genotype to cellular resilience, checkpoint activation, and DNA repair efficiency. These findings advance our understanding of how inter-individual variation shapes phototoxicity risk and contribute to the definition of human-relevant, mechanistic frameworks for UV-induced disease research.

The ability to generate melanocytes from iPSCs enables reproducible and scalable modeling of human pigmentation biology [13,14,15]. Our data confirm that differentiation yields mature melanocytes expressing canonical lineage markers (MITF, TYR, PMEL, MelanA, HMB45), with pigmentation intensity reflecting the underlying MC1R genotype. The observed range of UV survival among donor-derived lines directly correlated with their sun-sensitivity variable, validating this system as a quantitative surrogate for in vivo responses. These consistencies further support the translational relevance of our model and its capacity to recapitulate patient-specific UV responses observed in vivo. These findings are consistent with previous reports showing that MC1R signaling regulates melanocyte survival and DNA-repair capacity through cAMP-dependent and p53-mediated pathways [9,33]. Importantly, the differential UV survival patterns observed among iPSC-derived melanocytes parallel those previously reported in primary melanocytes and clinical cohorts stratified by MC1R genotype and pigmentation phenotype [7,9,29,34].

Our data show that the UV-sensitive Leeds3 melanocytes exhibit higher total melanin content than the more UV-resistant Leeds4 cells. This observation underscores that total melanin abundance alone is not a reliable predictor of UV protection. Instead, MC1R genotype critically influences melanocyte UV resilience by modulating receptor signaling, redox balance, and DNA damage responses. Loss-of-function MC1R variants, such as those present in Leeds3, are known to favor pheomelanin production, which is less photoprotective and can promote oxidative stress upon UV exposure. In contrast, functional MC1R signaling enhances cAMP-dependent activation of p53 and nucleotide excision repair pathways independently of pigment quantity. Thus, MC1R variants effectively “prime” melanocytes for differential stress responses, decoupling melanin content from UV tolerance.

We acknowledge that UV sensitivity is a multifactorial trait and that genetic modifiers beyond MC1R, including variants affecting DNA damage response pathways (e.g., ATM or ATR), may contribute to inter-individual differences. While comprehensive genotyping of additional DNA repair genes was not performed in this study, our results demonstrate that the MC1R genotype provides a strong and biologically relevant framework for stratifying UV response in this iPSC-based model.

UV-induced DNA damage is primarily manifested as cyclobutane pyrimidine dimers (CPDs) and 6-4 photoproducts [1,2]. Efficient repair of these lesions through nucleotide excision repair (NER) prevents mutagenesis and photocarcinogenesis. In our model, UV-sensitive melanocytes displayed CPD-associated signaling patterns consistent with delayed checkpoint resolution. Persistent activation of γH2AX, pATM, and pCHK1 in these lines mirrors the phenotype of MC1R-deficient cells, which show diminished ATR/p53 signaling and inefficient GADD45-mediated repair [34,35]. This phenotype parallels recent findings in human skin-derived models, where prolonged γH2AX retention and checkpoint persistence strongly correlate with apoptotic commitment [30,31]. Together, these results underscore the interplay between pigmentation genotype and DDR fidelity as a basis for differential UV outcomes.

Beyond direct DNA lesions, UV exposure also generates reactive oxygen species (ROS) and lipid peroxidation products that amplify stress signaling through Nrf2 and MAPK pathways [22,23,36,37]. This oxidative component of UV injury modulates melanocyte fate by tipping the balance between adaptive and apoptotic outcomes. Our findings indicate that loss-of-function MC1R alleles, which impair cAMP/PKA signaling, result in prolonged ROS accumulation and inefficient NER activation. This aligns with previous studies linking *MC1R* variants to redox imbalance, mitochondrial stress, and defective antioxidant responses in human skin [16,34,38]. Thus, pigmentation genotype integrates both pigment chemistry and redox regulation to define the cellular threshold for UV tolerance.

In this broader context, UV-induced stress responses in melanocytes extend beyond direct DNA damage and include activation of innate immune and interferon-regulated pathways. While these responses have been extensively studied in immune cells and melanoma, their contribution to physiological UV responses in normal melanocytes remains less well defined. Interferon-stimulated genes, in particular, have emerged as modulators of cellular stress signaling and apoptotic thresholds following genotoxic insults.

Importantly, MX2 was investigated within the same genotype-informed framework established for MC1R-associated UV responses, allowing integration of pigmentation genotype with interferon-related stress signaling rather than representing an independent discovery arm of the study. An important observation of this study is the association of MX2 expression with UV-induced stress signaling signatures in normal melanocytes. While MX2 was previously characterized in melanoma as an interferon-inducible factor linked to cell-cycle arrest and immune signaling [22,23,24], our data reveal that its expression also amplifies p53/p38 activation under UV stress independently of AKT. This suggests that MX2 integrates stress and apoptotic signaling pathways beyond its canonical antiviral or IFN-regulated functions [22,39,40]. Elevated MX2 in UV-sensitive iPSC-derived melanocytes was accompanied by increased caspase activation and reduced viability, highlighting its potential role in reinforcing damage-induced checkpoint responses. Nevertheless, while our results establish a clear trend between MX2 upregulation and enhanced apoptosis, the causal role of MX2 in regulating UV-induced cell death remains to be functionally validated. Future studies employing targeted MX2 knockdown or CRISPR-mediated gene editing in normal human melanocytes will be essential to confirm its direct mechanistic contribution. Such approaches will help delineate whether MX2 acts as a driver or amplifier of p53/p38-dependent apoptotic signaling.

Mechanistically, MX2 may influence apoptotic signaling by regulating pro-apoptotic factors such as XAF1 and modulating STAT1 phosphorylation, as demonstrated in melanoma cells under interferon stimulation [22]. Although direct links to p53 stabilization or mitochondrial apoptotic pathways remain to be elucidated, its consistency with UV sensitivity supports the notion that innate immune effectors can cross-regulate DDR and pigmentation pathways. This expands the physiological scope of MX2 from an interferon effector in melanoma to a UV response modulator in normal melanocytes, integrating genotype, stress signaling, and DNA repair capacity. In this context, sustained MX2 activity may contribute to apoptotic clearance of damaged melanocytes through regulation of pro-apoptotic factors such as XAF1 and modulation of STAT1 phosphorylation [22]. Although similar checkpoint persistence mechanisms have been reported in other cell types, including fibroblasts and keratinocytes [41], their relevance to MX2 function in melanocytes remains to be confirmed.

Our findings also underscore how MC1R-driven pigment chemistry impacts ROS buffering and DDR activation. Eumelanin-rich cells scavenge radicals and efficiently couple cAMP-PKA signaling to p53-mediated repair, while pheomelanin-rich melanocytes generate additional ROS under UV exposure, enhancing mutagenic load [34,38]. This concept aligns with recent findings demonstrating that MC1R/MITF-driven melanogenic capacity modulates UV-induced stress signaling thresholds in human melanocytes [29]. The convergence of these redox and signaling differences with MX2 activity defines a spectrum of UV resilience across individuals. Notably, this framework integrates pigmentation genotype, DDR dynamics, and apoptosis thresholds as co-determinants of photoprotection.

From a broader mechanistic perspective, UV stress response in melanocytes exemplifies the integration of immune signaling, pigment biochemistry, and checkpoint control. The identification of MX2 as a regulator of XAF1 and STAT1 phosphorylation provides a potential link between interferon-regulated networks and stress-induced apoptotic signaling in melanoma cells [22].

Similar cross-talk between interferon-stimulated genes and DNA damage response pathways has been described in pancreatic islet cells under autoimmune stress, suggesting broader roles for interferons in cellular stress adaptation [42].

Our data extend this concept to melanocytes, positioning MX2 at the intersection of antiviral defense and photoprotection biology. Finally, this iPSC-based system provides a flexible experimental framework to investigate patient-specific UV responses under standardized conditions. Unlike conventional melanoma or keratinocyte models, iPSC-derived melanocytes maintain the donor’s genomic integrity and pigmentation genotype, enabling controlled studies of genetic and pharmacologic modifiers of UV sensitivity [13,15]. As regulatory agencies emphasize new approach methodologies (NAMs) for mechanistic toxicology, this model supports translational research in photoprotection, pigmentation disorders, and UV-induced disease susceptibility [43]. Recent advances in NAMs have demonstrated the utility of stem cell-derived skin models for phototoxicity testing, including pigmentation-related responses [44]. Integration of multi-omics profiling, high-content imaging, and predictive modeling could further refine these systems for NAM-based hazard assessment and personalized photoprotection strategies [45].

From a regulatory standpoint, our iPSC-based approach aligns with the principles of new approach methodologies (NAMs) endorsed by the OECD for non-animal safety testing and mechanistic toxicology (OECD Test Guidelines TG 432, TG 497). Incorporating genetically defined human cell systems into NAM frameworks may improve the predictivity of phototoxicity assays and strengthen translational applications in personalized photoprotection.

This study was designed as a genotype-informed, mechanistic proof of concept rather than as a population-level association analysis. Accordingly, the number of iPSC-derived melanocyte lines examined in depth was limited, and the findings should be interpreted within this experimental scope. While the observed differences in UV response are consistent with known effects of MC1R variants on melanocyte biology, they do not establish a comprehensive or causal relationship between specific MC1R genotypes and UV sensitivity across populations. Future studies incorporating a larger number of genetically diverse iPSC lines will be required to generalize these findings and assess the contribution of additional genetic modifiers.

In particular, the limited number of sun-insensitive iPSC-derived melanocyte lines reflects the available donor material and reinforces that the present study was not designed to assess population-level genotype–phenotype associations.

Collectively, our results support an association between MX2 expression and amplification of UV-induced p53/p38 signaling dynamics in a genotype-dependent manner, positioning it as a potential molecular link between pigmentation, stress responses, and DNA repair efficiency. This work establishes iPSC-derived melanocytes as a human-relevant tool for studying the mechanistic basis of UV sensitivity and opens new directions for developing personalized photoprotective and NAM-based translational approaches.

## 4. Materials and Methods

Cells and cell culture. Normal human melanocytes (NHM) were isolated as previously described [46] and cultured in 254CF media (Invitrogen, Carlsbad, CA, USA) supplemented with Human Melanocyte Growth Supplement-2 (Gibco, Grand Island, NY, USA), Calcium chloride (Gibco, Grand Island, NY, USA), 5 mL Pen/Strep (cat. No. DE17-603E, Lonza, Basel, Switzerland) and 10 ng/mL PMA. Twenty-four hours before narrowband UVB (nbUVB) exposure, GFP/MX2 expression in transduced NHM134 was induced with 500 ng/mL doxycycline treatment. Melanoma cells were cultured in RPMI 1640 medium (Bio Whittaker, Verviers, Belgium) supplemented with 5% fetal bovine serum (cat.no F7524, Sigma-Aldrich, St. Louis, MO, USA) and 2 mM L-glutamine (cat. No. 17-605E, Lonza, Basel, Switzerland). Human dermal fibroblasts were derived and established from primary melanoma patients (Leeds, UK) by skin punch biopsy as previously described [47]. Fibroblasts were cultured in DMEM containing 10% FBS. Human embryonal stem cells H1, purchased from WiCell (cat. No. WA01, WiCell (Madison, WI, USA.)), and derived iPSC lines were cultured with MEF feeders (cat. No. 6001G, GlobalStem, Rockville, MD, USA) on tissue culture plates coated with 0.1% gelatin solution (cat. No. ES-006-B, Millipore, Billerica, MA, USA) in stem cell culture media consisting of 400 mL DMEM/F-12 (cat. No. 31330-038, Invitrogen (Carlsbad, CA, USA)), 100 mL KnockOut Serum Replacement (KOSR) (cat. No. 10828-028, Invitrogen (Carlsbad, CA, USA)), 5 mL Pen/Strep (cat. No. DE17-603E; Lonza, Basel, Switzerland), 2 mM L-glutamine (cat. No. 17-605E, Lonza, Basel, Switzerland), 5 mL NEAA (cat. No. 11140035, Invitrogen (Carlsbad, CA, USA)), 100 mM 2-mercaptoethanol (cat. No. 31350010, Invitrogen (Carlsbad, CA, USA)) and 10 ng/mL bFGF (cat. No. PHG0023, Invitrogen (Carlsbad, CA, USA)). All cells were maintained at 37 °C in a humidified 5% CO_2_ atmosphere.

Generation of Human-Induced Pluripotent Stem Cells from human dermal fibroblasts.Fibroblasts were reprogrammed to iPSCs using the CytoTune™-iPS Reprogramming kit (cat. No. A13780, Life Technologies, Carlsbad, CA, USA) and following the manufacturer’s protocol. This method is based on the Sendai reprogramming platform, which follows the non-integrating OSKM Sendai system [28]. Selected iPSC colonies were characterized for pluripotency and trilineage differentiation potential using TaqMan^®^ hPSC Scorecard™ (cat No. A15876, ThermoFisher Scientific, Waltham, MA, USA). iPSC clones with optimal Scorcard results were also validated by flow cytometry for pluripotency markers and selected for melanocyte differentiation.

Embryoid Body (EB) formation. For embryoid body formation, selected iPSC clones and H1 cells were transferred to low attachment plates and cultured in EB medium, containing DMEM/F-12 (ref. 31330-038, Invitrogen (Carlsbad, CA, USA)) supplemented with 20% KnockOut Serum Replacement (cat. No. 10828-028, Gibco, Grand Island, NY, USA), 50 U/mL Pen/Strep (cat. No. DE17-603E, Lonza, Basel, Switzerland), 2 mM L-Glutamine (cat. No. 17-605E, Lonza, Basel, Switzerland), 1% MEM NEAA (cat. No. 11140035, Invitrogen (Carlsbad, CA, USA)) and 0.2% 2-mercaptoethanol (cat. No. 31350010, Invitrogen (Carlsbad, CA, USA)). EBs were formed and maintained in the EB medium for up to 6 days, after which melanocyte differentiation was initiated.

Melanocyte differentiation. IPSC/H1 differentiation into melanocytes was performed as described previously [48]. In brief, embryoid bodies were resuspended in melanocyte induction media (Supplementary Table S3) and plated on human fibronectin-coated 6-well plates. The media was changed every 2 days for up to 5 weeks. When bipolar and/or dendritic pigmented cells were observed in the wells, they were mechanically dissociated and maintained in melanocyte-supporting medium. Isolated cells were tested for pigmentation and melanocyte differentiation markers.

Flow Cytometry: To assess iPSC for the presence of pluripotency markers, after fixation, cells were incubated with SSEA4 (cat. No. 09-0006, Stemgent, Cambridge, MA, USA) and TRA-1-60 (cat. No. MAB-4360, Merck-Millipore, Darmstadt, Germany) antibodies, followed by incubation with secondary anti-mouse Alexa Fluor 647 (cat. No. A32728, Invitrogen (Carlsbad, CA, USA)) and anti-rabbit PE (cat. No. 12-4739-81, Invitrogen (Carlsbad, CA, USA)).

For cell-cycle analysis, 3 × 10^5^ melanocytes per well were seeded into 6-well plates 24 h before nbUVB irradiation. Twenty-four hours after UV treatment, cells were harvested by trypsinization, washed twice in ice-cold PBS, and fixed by resuspending cell pellets in 1 mL 100% ice-cold methanol. Fixated cells were stained with a ready-to-use DNA Labeling Solution (Cytognos, Salamanca, Spain, cat.no. CYT-PIR-25). Stained cells were analyzed on a BD FACSCalibur^TM^ Flow cytometer (BD Biosciences, San Jose, CA, USA). Data were analyzed with FlowJo v.7.6.1 software (Treestar Inc., Ashland, OR, USA).

Immunohistochemistry: Sections from formalin-fixed paraffin-embedded 3D reconstructs were immunostained using the Dako EnVision™+ system (K8012, Dako Cooperation, Carpinteria, CA, USA) and Dako Autostainer. Deparaffinization, rehydration, and target retrieval were performed in a Dako PT link and EnVision™ FLEX Target Retrieval Solution, low pH (citrate buffer, pH 6.1). Endogenous peroxidase was blocked using Dako blocking reagent for 5 min, followed by incubation with rabbit monoclonal cleaved caspase 3 antibody (#9664; Cell Signaling (Danvers, MA, USA)) diluted 1:500 for 30 min. Thereafter, the sections were incubated with Dako EnVision™ FLEX+ rabbit linker for 15 min, followed by incubation with Dako EnVision™ FLEX/HRP for an additional 30 min. For visualization of staining, the sections were treated with 3,3′-diaminobenzidine tetrahydrochloride (DAB), counterstained with hematoxylin, and mounted in Richard-Allan Scientific™ Cytoseal™ XYL (Thermo Scientific, Waltham, MA, USA). Negative controls included the substitution of the polyclonal primary antibody with normal rabbit IgG protein at the same concentration.

Immunofluorescence: Melanocytes were seeded onto 6-well plates with glass bottom at a density of 3 × 10^5^ cells per well. For fixation, melanocytes were overlaid with 100% ice-cold methanol and allowed to fix for 15 min at −20 °C. Methanol was aspirated, and cells were rinsed three times with PBS. To avoid non-specific antibody binding, cells were incubated with blocking solution (5% goat serum (cat. No. ab7481, Abcam (Cambridge, UK) in PBS) for 1 h at room temperature. The blocking solution was aspirated, and cells were stained with primary antibodies overnight at 4 °C, diluted to 1:200 and specific for HMB45 (cat. No. ab787, Abcam, Cambridge, UK) and MelanA (cat. No. ab51061, Abcam, Cambridge, UK). Cells were then rinsed three times with PBS, and secondary antibody Alexa Fluor 488 (Invitrogen, Carlsbad, CA, USA) diluted to 1:500 was added for 1 h at room temperature. After three washes, cells were stained with DAPI and examined using a Leica DMI600 B inverted fluorescence microscope (Leica, Wetzlar, Germany).

Quantitative real-time Reverse-Transcription PCR: RT-qPCR reactions were performed as previously described [23] using TaqMan Gene Expression Assays: MITF (Hs01117294_m1), TYR (Hs00165976_m1), and PMEL (Hs00173854_m1). Transcript expression levels were normalized to a housekeeping gene, beta-glucuronidase (GUSB; Hs99999908_m1). Expression of endogenous pluripotency markers was assessed using the TaqMan iPSC Sendai Detection Kit (cat. No. A13640, Life Technologies, Carlsbad, CA, USA) according to the manufacturer’s protocol.

Incucyte growth rate assessment: For cell growth determination, melanocytes were seeded into 96-well plates at a density of 10,000 cells per well. Cell proliferation was assessed using a confluence assay with the IncuCyte^TM^ FLR (Essen Instruments, Ann Arbor, MI, USA) live-cell imaging system. Cell growth rate was determined by normalizing cell confluence at a given time to the corresponding initial time point. Data are presented as the mean ± SE from three independent experiments.

Viability assay: For viability assays, 10,000 melanocytes and 5000 melanoma cells were seeded into 96-well plates a day prior to nbUVB irradiation. Seventy-two hours after the nbUVB insult, cell viability was determined using CellTiterGlo luminescent viability assay (cat. No G7570) purchased from Promega (Madison, WI, USA) and following the manufacturer’s protocol. Luminescence was recorded with a Fluoroskan Ascent FL luminometer (Thermo Fisher Scientific, Waltham, MA, USA).

DNA extraction: A total of 3 × 10^5^ melanocytes per well were seeded into 6-well plates 24 h before nbUVB irradiation with 1.5 J/cm^2^. At different time points after irradiation, genomic DNA was extracted using a DNA extraction kit according to the manufacturer’s protocol (ref. 740952.250, Macherey-Nagel, Düren, Germany) and stored at −20 °C until use.

Quantification of CPDs by ELISA: Genomic DNA (gDNA) was denatured by incubating the sample at 95 °C for 10 min, followed by immediate cooling on ice for an additional 10 min. gDNA was diluted in DNA coating solution (cat. No. 17250, Thermo Scientific (Waltham, MA, USA) to yield a final concentration of 2 ng/µL and 50 µL of each sample was loaded per well on a DNA-BIND 96-well plate (ref. 2525, Sigma-Aldrich, St. Louis, MO, USA) in duplicate. Incubation lasted overnight at 37 °C, then each well was washed 5 times with 150 μL PBST (0.05% Tween-20 (cat. No. P1379, Sigma-Aldrich, St. Louis, MO, USA) in 1× PBS). To prevent non-specific antibody binding, 100 μL of 2.5% FBS (cat. No F7524, Sigma-Aldrich, St. Louis, MO, USA) in 1× PBS was loaded into each well for 1 h at 37 °C. After 5 washes with 150 μL PBST, 100 μL per well of 1:1000 diluted anti-CPD antibody (clone TDM2, cat. No CAC-NM-DND-001, Cosmo Bio USA, Carlsbad, CA, USA) in 2.5% FBS was loaded and incubation lasted for 1 h at 37 °C. After 5 washes with 150 μL PBST, the plate was incubated with 100 μL per well of 1:1000 diluted secondary HRP conjugated anti-mouse IgG(H + L) antibody (cat. no. W4021, Promega, Madison, WI, USA). The plate was washed 5 times with 150 μL PBST and incubated with 100 μL 1-Step™ Turbo TMB-ELISA substrate solution (cat. No. 34022, Thermo Scientifi, Waltham, MA, USA) for 30 min at room temperature. Reaction was stopped with 100 μL ELISA Stop Solution (cat. No. SS04, Invitrogen, Carlsbad, CA, USA) and absorbance was measured at 450 nm with a reference at 620 nm on the Multiskan FC microplate photometer (Thermo Scientific, Waltham, MA, USA).

Melanin measurements: A pellet of 10^6^ melanocytes was dissolved in 1 mL Soluene-350 in the presence of 10% dH_2_O and heated at 60 °C for 3 h with agitation. Absorbance was measured at 500 nm using a Perkin Elmer spectrometer (PerkinElmer, Waltham, MA, USA). To convert the absorbance value to the total melanin amounts, a standard calibration curve of synthetic melanin (Sigma-Aldrich, St. Louis, MO, USA) was generated. Three independent experiments to evaluate melanin contents were performed for each melanocyte culture.

Generation of stable lines overexpressing MX2 and GFP: Cell cultures were established as described in a publication [23].

Immunoblotting: For immunoblotting analyses, melanocytes were seeded at 3 × 10^5^ cells per 6-well plate (BD Falcon, Franklin Lakes, NJ, USA). 2, 6 and 24 h after nbUVB treatments, cells were harvested and analyzed for protein expression as previously done [46]. Primary antibodies used in a study: Cell Signaling (Danvers, MA, USA): PARP (#9532) 1:1000, Caspase 3 (#9662) 1:5000, Cleaved Caspase 3 (#9664) 1:1000, GAPDH (#2188) 1:2000, phospho-ATM s1981 (#13050) 1:1000, ATM (#2873) 1:1000, phospho-CHK1 s345 (#2341) 1:1000, CHK1 (#2345) 1:1000, phospho-CHK2 t68 (#2661) 1:1000, CHK2 (#3440) 1:1000, γH2AX (#9718) 1:1000, phospho-AKT s473 (#4060) 1:2000, AKT (#9272) 1:2000, phospho-H3 s10 (#9701) 1:1000, H3 (#4499) 1:3000, β-actin (#4967), phospho-p38 MAPK thr180/tyr182 (#4631) 1:1000; Santa Cruz Biotechnology (Dallas, TX, USA): GADD45α (sc-6850) 1:1000, p53 (sc-126); Novus Biologicals (Littleton, CO, USA): MX2 (NBP1-81018) 1:2000; Abcam (Cambridge, UK): phospho-CDK1 y15 (ab47594) 1:2000. Secondary antibodies were purchased from Promega (Madison, WI, USA): anti-rabbit (W4011) 1:2000, anti-mouse (W4021) 1:2000. Immunoblots displayed in figures are representative of at least two independent experiments.

3D skin reconstructs: Skin reconstructs were generated as described in reference [49]. Briefly, each insert of tissue culture trays was coated with 1 mL mixture containing 98 µL 10× EMEM media, 8.4 µL 200 mM L-glutamine, 100 µL FBS, 760 µL collagen I (cat. No. #5005, Advanced Biomatrix, Carlsbad, CA, USA) and 20 µL 7.5% sodium bicarbonate and incubated at 37 °C for 1 h. Afterward, 7.5 × 10^4^ fibroblasts in 250 µL DMEM containing 10% FBS were mixed with 274 µL 10× EMEM media, 25 µL 200 mM glutamine, 308 µL FBS, 2320 µL collagen I, and 58 µL 7.5% sodium bicarbonate and pipetted on each insert. After solidification, 2mL of DMEM containing 10% FBS was added inside, and 10 mL was added to the outside of the insert. After four days of incubation at 37 °C in a 5% CO_2_ tissue culture incubator, a mixture of iPSC-derived melanocytes and keratinocytes was added on top of the dermal reconstructs (ratio 1:5 melanocytes to keratinocytes) in skin reconstruct medium: keratinocyte serum-free medium (cat. No. 17005042, Gibco, Grand Island, NY, USA) supplemented with 60 µg/mL bovine pituitary extract, 10 ng/mL SCF, 4.5 ng/mL bFGF, 100 nM ET3 and 1 ng/mL epidermal growth factor (EGF). After two days, the EGF concentration was lowered to 0.2 ng/mL, and after two additional days, the surface of the reconstructed tissue was exposed to air for 1.5 weeks, while high-calcium (2.4 mM) medium was added only to the outside of the insert. For the UV irradiation experiment, media outside of the insert were replaced with PBS prior to the exposure, and skin reconstructs were harvested 48 h after irradiation, fixed in 10% neutral buffered formalin for 3 h, and assessed for activated caspase 3 by immunohistochemistry.

RPPA: The samples were prepared as previously described [50]. RPPA was performed by the MD Anderson Center RPPA core facility (Houston, TX, USA) as previously described [51]. Unsupervised or supervised hierarchical clustering was performed on normalized log2 median-centered protein values using the Cluster 3.0 software, with centered correlation and complete linkage. Results were visualized using Java TreeView 3.0 software. For UV time-course analysis, normalized Log2 values were median-centered relative to the untreated controls. K-means clustering using Euclidean distance measure on 20 clusters (identified in unsupervised hierarchical clustering), run for 100 iterations, was performed using Cluster 3.0 and visualized with Java TreeView. Clusters with variance greater than 0.10 across time points were selected for biological process analysis using Ingenuity Pathway Analysis (Qiagen, Hilden, Germany).

nbUVB irradiation: Cell plates with a culture dish lid were irradiated at room temperature from above with the narrowband UVB (nbUVB) source consisting of four 20-watt nbUVB lamps with an output peak at 311 nm within the range 305–315 nm and a fluence rate of 0.83 mW/cm^2^. (TL20W/01, Philips, Holland, The Netherlands). UV output was measured before each irradiation session with a calibrated thermopile detector coupled to an optic power meter Gentec-EO SOLO2 (Gentec EO, Quebec, QC, Canada). IR interference was removed by an IR filter (XLP12 model). Prior to irradiation, the cell medium was removed from the wells and substituted with DPBS (cat. No. D8662, Sigma-Aldrich, St. Louis, MO, USA) supplemented with 0.1% glucose. After exposure, DPBS was discarded and cells in culturing media were returned to the incubator and harvested at different time points for corresponding assays.

Statistical analyses: Statistical analysis was performed applying SPSS package Version 18 (SPSS Inc., Chicago, IL, USA). A two-tailed paired Student’s *t*-test was used for the evaluation of in vitro results. A *p*-value of less than 0.05 was considered statistically significant.

## 5. Conclusions

This study supports the use of iPSC-derived melanocytes generated from patient fibroblasts carrying distinct MC1R pigmentation variants as a genetically defined human model for studying UV-induced cellular responses. Our results support the hypothesis that UV response differences associated with donor background can be faithfully modeled using iPSC-derived melanocytes in this study. Consistent with this framework, donor-associated differences in DNA damage signaling, checkpoint activation, and repair kinetics were observed across melanocyte lines with distinct pigmentation genotypes.

At a mechanistic level, our findings support MX2 as an important modulator of UV-induced p53/p38 signaling and apoptosis in melanocytes, acting independently of AKT and extending its functional relevance beyond interferon-mediated roles previously described in melanoma. These observations suggest that MX2 contributes to the cellular stress response following UV exposure in normal human melanocytes.

Collectively, this work provides a human-relevant and reproducible experimental platform for investigating molecular determinants of UV-induced stress responses and disease susceptibility. In this context, iPSC-derived melanocyte systems align well with emerging new approach methodologies (NAMs) for mechanistic toxicology and offer opportunities for translational studies in pigmentation biology, DNA repair, and photoprotection.

## Data Availability

The data supporting the findings of this study are included within the article and its Supplementary Materials. Additional datasets generated and/or analyzed during the current study are available from the corresponding authors upon reasonable request.

## Supplementary Materials

The following supporting information can be downloaded at: https://www.mdpi.com/article/10.3390/ijms27062617/s1.

## Abbreviations

The following abbreviations are used in this manuscript:

**Abbreviation**	**Definition**
α-MSH	Alpha-melanocyte-stimulating hormone
AKT	Protein kinase B
ATM	Ataxia telangiectasia mutated
ATR	Ataxia telangiectasia and Rad3-related
bFGF	Basic fibroblast growth factor
CDK1	Cyclin-dependent kinase 1
cAMP	Cyclic adenosine monophosphate
CHK1	Checkpoint kinase 1
CHK2	Checkpoint kinase 2
CPD	Cyclobutane pyrimidine dimer
DDR	DNA damage response
DMEM	Dulbecco’s modified Eagle’s medium
DPBS	Dulbecco’s phosphate-buffered saline
EB	Embryoid body
EGF	Epidermal growth factor
FACS	Fluorescence-activated cell sorting
FBS	Fetal bovine serum
GADD45α	Growth arrest and DNA damage-inducible protein alpha
GFP	Green fluorescent protein
GUSB	Beta-glucuronidase
γH2AX	Phosphorylated histone H2AX
HRP	Horseradish peroxidase
iPSC	Induced pluripotent stem cell
IRDS	Interferon-related DNA damage resistance signature
MAPK	Mitogen-activated protein kinase
MC1R	Melanocortin 1 receptor
MITF	Microphthalmia-associated transcription factor
MX2	Myxovirus resistance protein 2
NAMs	New Approach Methodologies
nbUVB	Narrowband ultraviolet B
NER	Nucleotide excision repair
NHM	Normal human melanocytes
OSKM	OCT4, SOX2, KLF4, and c-MYC
PARP	Poly(ADP-ribose) polymerase
PBS	Phosphate-buffered saline
PKA	Protein kinase A
PMA	Phorbol 12-myristate 13-acetate
PMEL	Premelanosome protein
QC	Quality control
ROS	Reactive oxygen species
RPPA	Reverse phase protein array
RT-qPCR	Quantitative reverse-transcription polymerase chain reaction
SCF	Stem cell factor
SSEA-4	Stage-specific embryonic antigen 4
STAT1	Signal transducer and activator of transcription 1
TG	Test guideline
TYR	Tyrosinase
UV	Ultraviolet
XAF1	XIAP-associated factor 1
