# Supplementary material for: Genotype-Encoded UV Sensitivity in iPSC-Derived Human Melanocytes Reveals MX2 as a Physiological Amplifier of p53/p38-Mediated DNA Damage Signaling
Source: Int J Mol Sci. 2026 Mar 12;27(6):2617. doi: 10.3390/ijms27062617 (PMC13027049; doi:10.3390/ijms27062617)
Supplement: Supplementary file 1 [file ijms-27-02617-s001.zip › Supplementary Figure Legends.pdf]

Supplementary Figure S1. Validation of pluripotency in iPSCs prior to melanocyte differentiation. Pluripotent stem cell identity was verified before lineage induction to ensure genomic and transcriptional integrity of reprogrammed donor lines. **(A)** Immunofluorescence staining for TRA-1-60 and SSEA-4 (green) with DAPI nuclear counterstaining (blue) confirms robust surface expression of canonical pluripotency markers in reprogrammed fibroblast-derived iPSCs. **(B)** qRT-PCR analysis demonstrates high transcript levels of *Nanog*, *Oct4*, and *Sox2*, validating the maintenance of core pluripotency circuitry. The combination of surface and transcriptional markers establishes the fidelity of reprogramming across all donor lines, ensuring equivalent developmental potential prior to differentiation into the melanocytic lineage. Scale bar: 50 µm. Data represent mean ± SEM of three independent experiments.

Supplementary Figure S2. Immunofluorescence validation of canonical melanocytic markers (S100, Mel-5, HMB45) in iPSC-derived melanocytes. Representative immunofluorescence of iPSC-derived melanocytes showing expression of canonical melanocyte markers S100, Mel-5, and HMB45, including dual-stain combinations (HMB45/S100; Mel-5/S100). Although only representative images are shown, all iPSC-derived melanocyte lines used in this study exhibited the same marker expression patterns, corroborating melanocytic identity and complementing the lineage-marker validation shown in Figure 1.

Supplementary Figure S3. Functional modulation of MX2 alters UV-induced apoptosis in melanoma cells. To assess the functional contribution of MX2 to UV-induced stress signaling, melanoma cells were engineered to express either GFP or MX2-GFP. **(A)** Immunoblot analysis confirms efficient MX2 overexpression relative to GFP controls, with consistent protein levels across replicates. **(B)** Quantification of cell viability 48 h after nbUVB exposure (1.5 J/cm^2) shows significantly reduced survival in MX2-overexpressing cells, consistent with heightened apoptotic engagement. Statistical significance was determined by one-way ANOVA followed by Tukey's multiple comparison test (* $p < 0.05$). Morphological analysis reveals increased cell rounding and detachment in MX2-GFP cultures, corroborating enhanced stress sensitivity. These data support a causal role for MX2 in amplifying apoptosis under UV stress, complementing the observations made in non-transformed melanocytes. Data represent mean ± SEM of three independent experiments.
