# Supplementary figures and images for: Genotype-Encoded UV Sensitivity in iPSC-Derived Human Melanocytes Reveals MX2 as a Physiological Amplifier of p53/p38-Mediated DNA Damage Signaling
Source: Int J Mol Sci. 2026 Mar 12;27(6):2617. doi: 10.3390/ijms27062617 (PMC13027049; doi:10.3390/ijms27062617)

**A**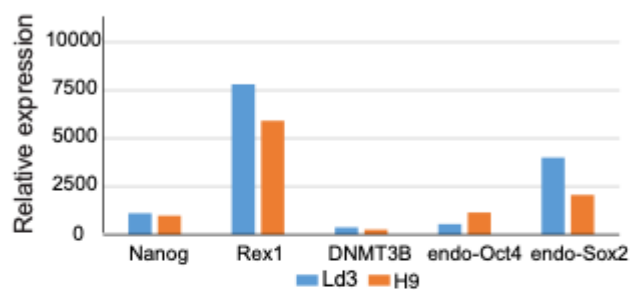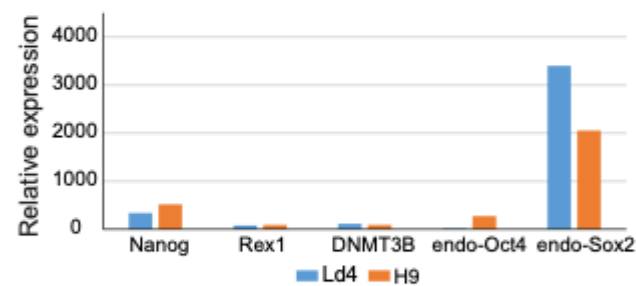**B**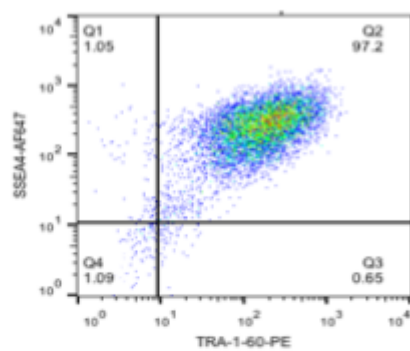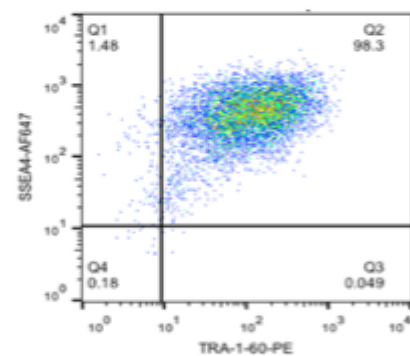

Supplement: Supplementary file 1 [file ijms-27-02617-s001.zip › Figure S1.pdf]

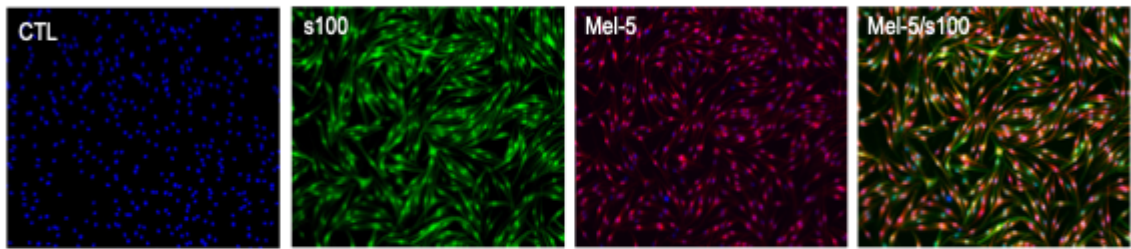

Supplement: Supplementary file 1 [file ijms-27-02617-s001.zip › Figure S2.pdf]

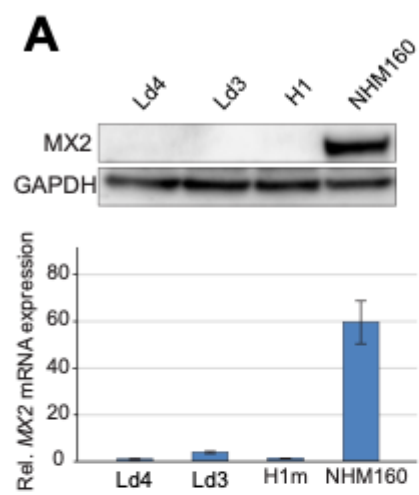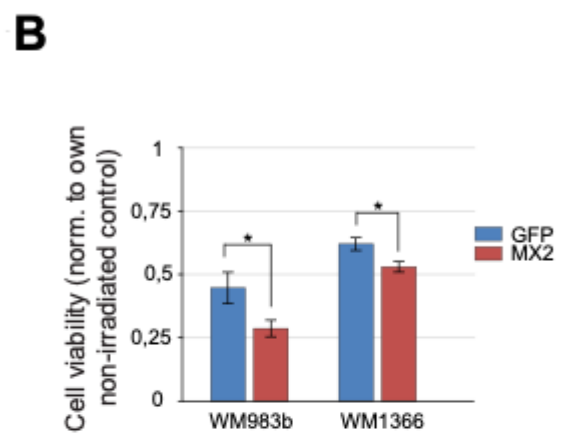

Supplement: Supplementary file 1 [file ijms-27-02617-s001.zip › Figure S3.pdf]
